# A Rare Case of Malignant Phyllodes Tumor of the Breast in a Patient With a History of Recurrent Fibroadenomas: A Case Report and Literature Review

**DOI:** 10.7759/cureus.99133

**Published:** 2025-12-13

**Authors:** Kendall A Vignaroli, Sharmila Raju, Michelle Lee, Angel Guan, Blaine Morton

**Affiliations:** 1 General Surgery, Arrowhead Regional Medical Center, Colton, USA; 2 General Surgery, Kaiser Permanente Fontana Medical Center, Fontana, USA

**Keywords:** brain metastasis, breast fibroadenoma, distant metastasis, lung metastasis, malignant phyllodes tumor, rare breast malignancy

## Abstract

The distinction between a fibroepithelial tumor and phyllodes tumor of the breast is clinically significant, as phyllodes tumors and fibroadenomas differ in their biological behavior and thus their management. Malignant phyllodes tumors are extremely aggressive, and most cases with metastasis are unresponsive to chemotherapy and carry a poor prognosis. Due to the rarity of phyllodes tumors and, in particular, malignant phyllodes, the literature evaluating this pathology and its outcomes is sparse; there is currently no evidence-based guideline available for its optimal management.

A 29-year-old female underwent an excisional biopsy of right breast fibroadenomas on two separate occasions over the course of five years. Upon a third recurrence, ultrasound noted features suspicious for malignancy, and core biopsy showed fibroadenoma. She underwent excisional biopsy of four masses, where pathology showed fibroepithelial lesion with necrosis in one mass, borderline phyllodes tumor in two masses, and a malignant phyllodes tumor in the fourth mass with positive margins. The patient underwent a right simple mastectomy, was found to have metastasis to the lung, liver, and brain nine months after mastectomy, and died 10 months after mastectomy at the age of 36 years. Given the known tumor heterogeneity and the overall diagnostic uncertainty involved in fibroepithelial masses and phyllodes tumors, it is imperative to maintain heightened suspicion and to utilize additional sampling methods when evaluating fibroepithelial lesions with high-risk features, including hypercellularity, necrosis, or recurrence. Additionally, there is a need for further research and definitive management guidelines for patients with borderline and malignant phyllodes tumors who have undergone mastectomy, as these patients still have a high risk of distant metastasis.

## Introduction

Phyllodes tumors (PTs) are observed in less than 1% of all breast tumors, and are distinguished from fibroadenomas only by subtle histologic differences [1]. However, the distinction between a fibroepithelial mass and a PT is clinically significant, as PTs and fibroadenomas differ in their biological behavior and thus their management.

PTs are subclassified as benign, borderline, and malignant as follows: benign tumors account for 60-75% of PTs, borderline tumors 12-26%, and malignant tumors 10-15%. This subclassification is based on the presence of histopathological features, including stromal cellularity, atypia, mitotic activity, stromal overgrowth, and tumor margins [1]. Because of their rarity, PTs can be mistaken for fibroadenomas on physical exam, thus delaying their workup and diagnosis, which can lead to larger tumors at the time of resection and worse overall outcomes [2]. Malignant PT is known to be extremely aggressive, with local recurrence rates reported between 15% and 40%, a distant metastasis rate of around 20%, and mortality rates reported between 50% and 100%, with time of death reportedly 1.0-41.1 months after distant metastasis diagnosis [3]. Furthermore, most cases of PTs with metastasis are unresponsive to chemotherapy and therefore carry a poor prognosis [3]. The most common site of distant metastasis is the lung, and the second most common site is in bone. PT generally metastasizes hematogenously; while 10-15% of cases involve clinical lymphadenopathy, these instances are usually thought to be due to reactive hyperplasia secondary to infection or tumor necrosis. As such, routine axillary dissection is not generally recommended in cases of malignant PTs [3].

Currently, the National Comprehensive Cancer Network guidelines suggest maintaining a high clinical suspicion for the presence of a PT if a breast lesion is palpable, rapidly growing, or has a size larger than 3 cm; these factors should raise suspicion for the presence of a PT even if ultrasound imaging is otherwise consistent with a diagnosis of fibroadenoma [4]. Due to the rarity of PTs, in particular, malignant PTs, literature evaluating this pathology and its outcomes are sparse; there is currently no true evidence-based guideline or universally accepted consensus available concerning optimal management of PTs. In the available literature, topics such as surgical margins and adjuvant therapy have been debated, and thus, recommendations are constantly changing. Currently, the National Comprehensive Cancer Network (NCCN) guidelines recommend excisional biopsy for lesions with core needle biopsy suspicious of benign phyllodes, and wide local excision with a margin of at least 1 cm without axillary staging for lesions with core needle biopsy suspicious of borderline or malignant phyllodes [4,5].

Due to the paucity of literature and the lack of strong evidence-based guidelines for managing malignant PTs, case reports provide valuable insight into their presentation, behavior, and outcomes following different management approaches. We present a case describing a patient with a five-year history of recurrent fibroadenomas in the right breast after multiple excisions who was ultimately diagnosed with a malignant PT, underwent right simple mastectomy, and was eventually found to have metastasis to the lung, liver, and brain nine months after mastectomy. This case underscores the need to maintain heightened suspicion and utilize additional sampling methods when evaluating fibroepithelial lesions with high-risk features, highlights the importance of providing close follow-up for patients with recurrent fibroadenomas, and emphasizes the need for further research and definitive management guidelines in the treatment of patients with borderline and malignant phyllodes tumors who have undergone mastectomy, as these patients still have a high risk of distant metastasis.

## Case presentation

**Figure 1:**
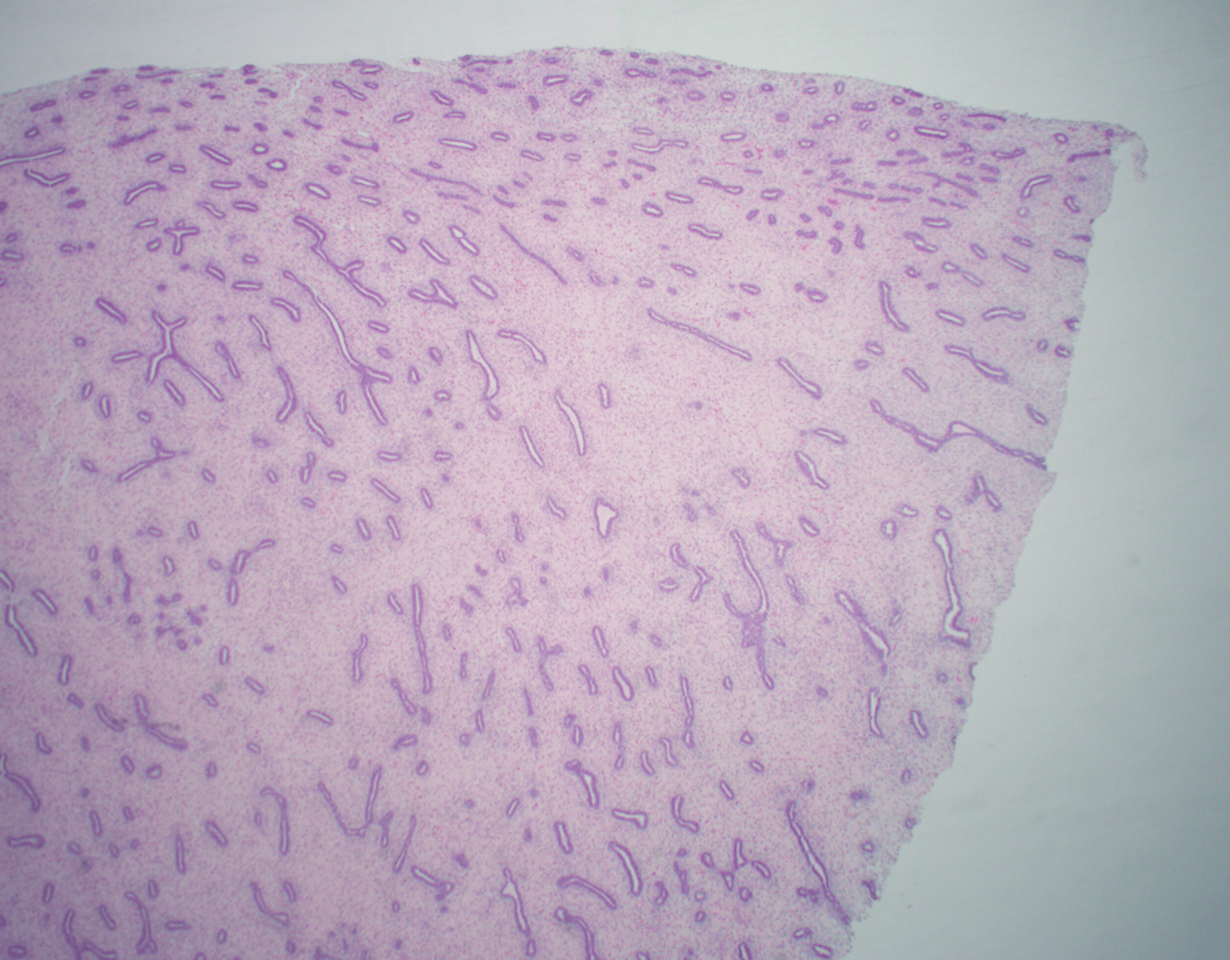
First fibroepithelial lesion excision (2018). Histology demonstrating a fibroepithelial lesion with an intracanalicular growth pattern.

A 29-year-old female with gastroesophageal reflux disease and hypertension had a family history notable for a paternal aunt who died of breast cancer in her 40s, a maternal grandfather with melanoma, and a maternal uncle with brain cancer. She presented in September 2018 with an enlarging mass in the right breast. A core needle biopsy revealed a fibroadenoma, and the patient underwent an excisional biopsy in December 2018, with final pathology describing a fibroadenoma with necrosis and a histiocytic reaction, and no evidence of malignancy (Figure 1).

**Figure 2:**
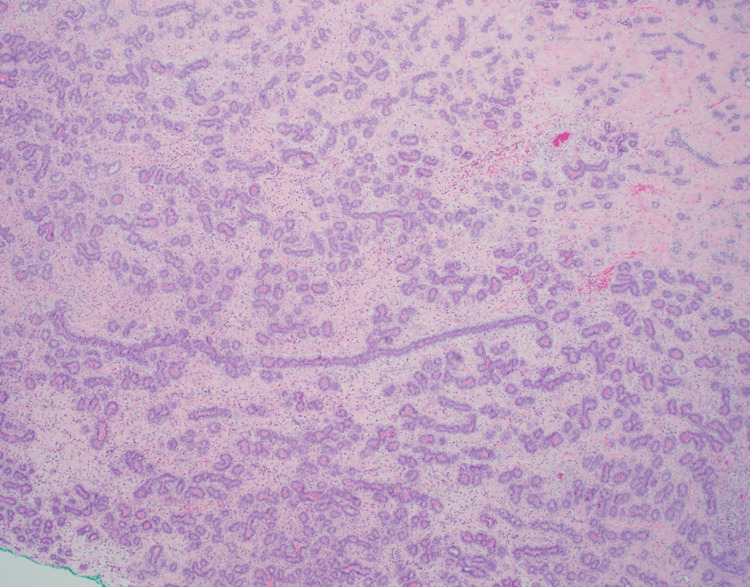
Second fibroepithelial lesion excision (2021). Histology demonstrating a fibroepithelial lesion with an intracanalicular growth pattern.

In December 2020, she returned to the clinic with a new right breast lump, and three well-defined masses were palpated in her right breast on examination. Biopsy of the largest lesion noted fibroadenoma with necrosis, and the patient underwent an excisional biopsy of two right breast masses in May 2021, both of which were determined to be fibroadenomas on pathology (Figure 2).

**Figure 3:**
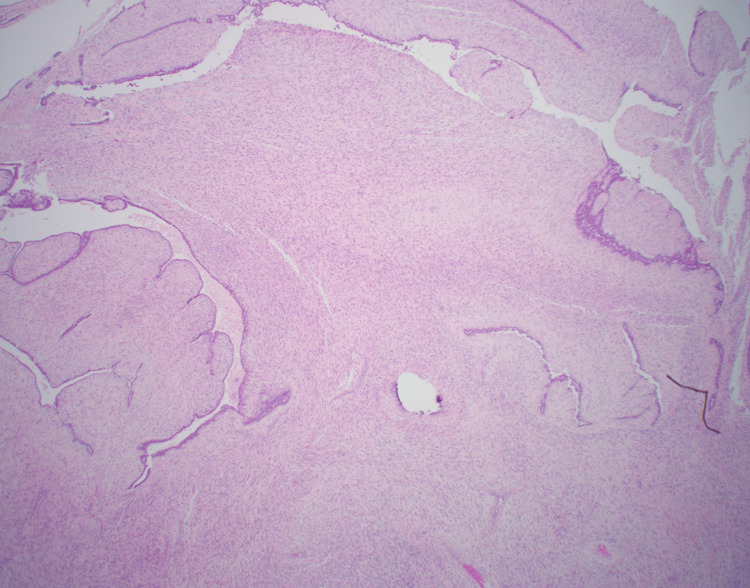
Malignant phyllodes tumor (2024). Histology showing a leaf-like architectural pattern characteristic of a malignant phyllodes tumor.

The patient returned in April 2023 at 35 years of age with the emergence of another right breast mass. The patient underwent a diagnostic mammogram, which revealed at least three masses measuring 6.7 cm total in the right breast and multiple associated lymph nodes in the right deep posterosuperior region. Subsequent diagnostic ultrasound noted three masses measuring 4.6 cm, 2.9 cm, and 1.9 cm in the right breast with features suspicious for malignancy. Core biopsy showed fibroadenoma and morphologically benign-appearing lymph nodes. The patient underwent excisional biopsy of four masses in June 2024, where pathology noted a fibroepithelial lesion with necrosis in one mass, borderline phyllodes tumors in two masses, and a 6.3 cm malignant phyllodes tumor in the fourth mass with positive superior, posterior, and lateral margins (Figure 3). A repeat ultrasound completed one month after the excision showed a 0.8x6.6x7.2 cm lobulated area in the right breast (thought likely to be residual phyllodes tumor with surrounding hematoma), as well as a 4.3 cm oval right axillary lymph node with eccentric cortical thickening. Due to the size of the mass on ultrasound and likely involvement of the retroareolar skin, the patient underwent a right simple mastectomy in August 2024. Though sentinel lymph node biopsy is not recommended for lumpectomies performed for phyllodes tumors, sentinel lymph node biopsy was completed during this operation because mastectomy is known to disrupt the lymphatic system, thus making sentinel lymph node biopsy infeasible in the future. Intraoperatively, frozen section pathology noted a negative sentinel lymph node and a 9.5 cm malignant phyllodes tumor with less than 1 cm margin to the skin along the inferior flap and no malignant heterologous elements identified. Additional inferior skin margin and lower pectoral margin were resected, which confirmed a negative margin resection on final pathology. The patient was staged as pT2, N0.

Post-operatively, the patient was evaluated by radiation oncologist, where she was informed that the role of adjuvant radiation therapy after surgery for phyllodes tumor of the breast is controversial. An extensive discussion was held regarding the National Comprehensive Cancer Network (NCCN) guideline recommendation to consider radiation after wide excision; however, a retrospective review was also discussed, which showed a decrease in local recurrence with adjuvant radiation therapy after a lumpectomy but not after a mastectomy for borderline and malignant phyllodes tumors [4,6]. Because the patient had undergone a mastectomy with negative margins and because she was planning on undergoing reconstruction, the joint decision was made to forgo adjuvant radiation therapy. The patient then had an extensive discussion with plastic surgery regarding reconstruction options, and after many months of thought, she made the decision to undergo a left prophylactic mastectomy and bilateral breast reconstruction with expander placement.

**Figure 4:**
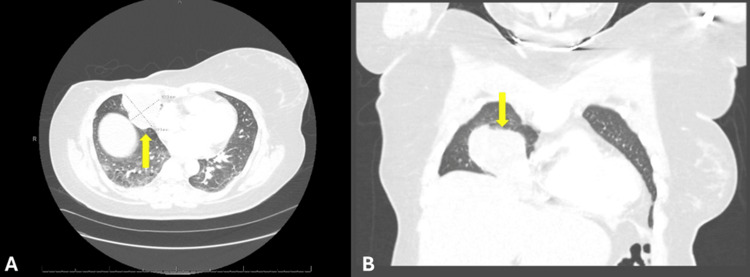
Cross-sectional computed tomography angiography (CTA) imaging of the chest and abdomen with IV contrast showing lung metastasis. (A) An axial view and (B) a coronal view displaying a 6.3×5.2 cm lesion (yellow arrow) abutting the anterior right chest wall.

Prior to her planned left prophylactic mastectomy and reconstructive surgery, the patient presented to the emergency department in May 2025 with right-sided pleuritic chest pain and hemoptysis, and CT angiography of the chest revealed a right middle lobe mass measuring 6.3x5.2 cm abutting the anterior right chest wall, suspicious for metastatic disease (Figures 4A, 4B).

**Figure 5:**
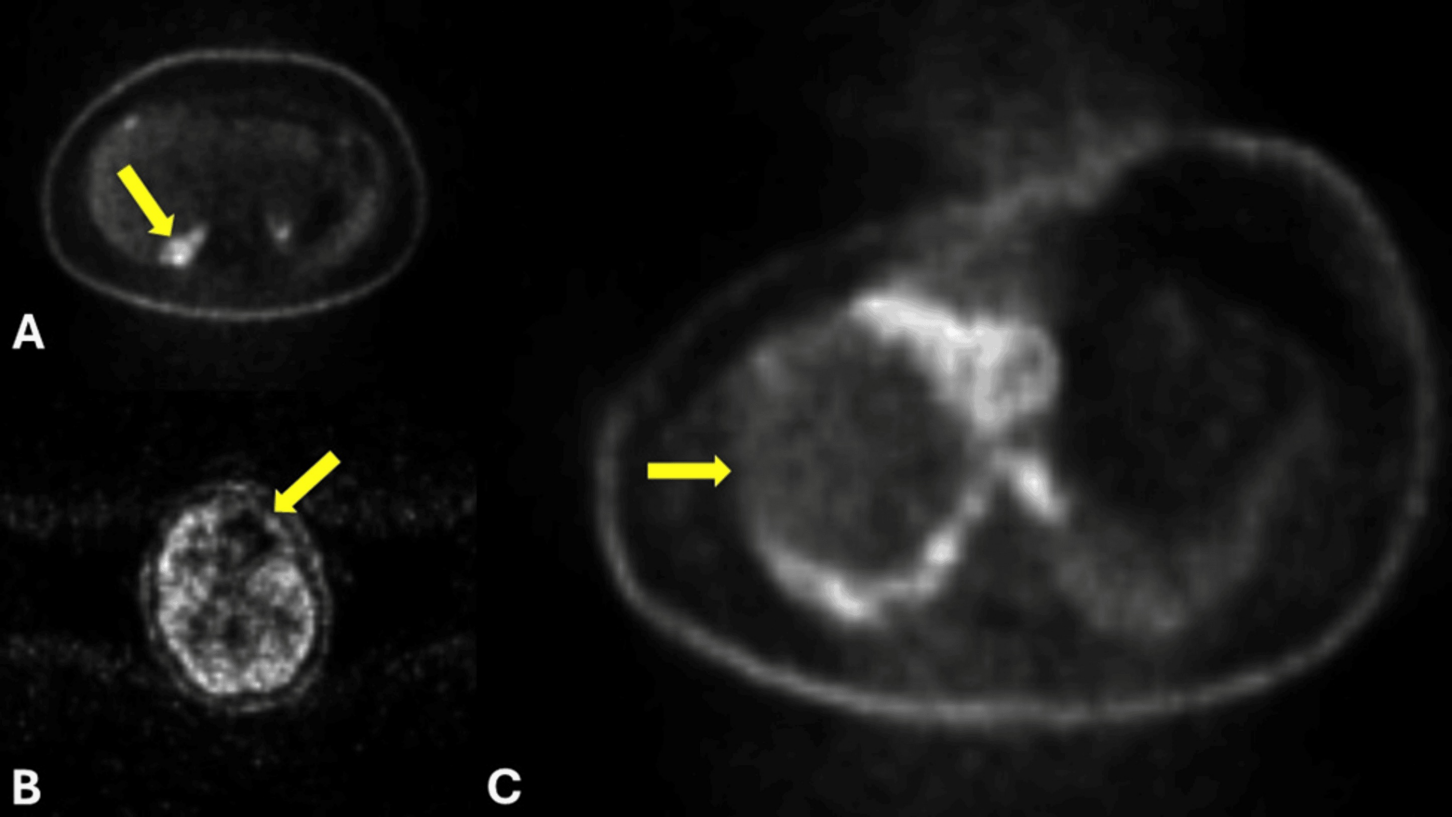
Positron emission tomography with metastasis. Cross-sectional positron emission tomography (PET) imaging with IV contrast showing (A) an axial view demonstrating hypermetabolic activity in the right hepatic lobe adjacent to the gallbladder (yellow arrow) (SUVmax 7.4), (B) an axial view demonstrating a large abnormal photopenic area in the left frontal lobe (yellow arrow), and (C) an axial view demonstrating hypermetabolic activity in the right middle lobe of the lung (yellow arrow), measuring 7.5×6.9×11 cm, with central necrosis and displacement of the mediastinum to the left (SUVmax 18). SUV: standardized uptake value

She underwent interventional radiology-guided biopsy of the mass in June 2025, which revealed an extensively necrotic spindle cell neoplasm suspicious for metastatic malignant phyllodes tumor. Her left mastectomy and breast reconstruction surgery was cancelled, and the patient was scheduled for right lung tumor resection in two weeks. However, one week later, at the end of June 2025, a positron emission tomography (PET) scan was completed, which was notable for hypermetabolic activity in the right hepatic lobe adjacent to the gallbladder, hypermetabolic activity in the right middle lobe lung mass measuring 7.5x6.9x11 cm with central necrosis and displacement of the mediastinum to the left, and right lung pleural thickening. The PET scan impression also noted that, though PET is generally limited for evaluation of intracranial disease, there was a large, abnormal, photopenic area in the left frontal lobe, and the recommendation was made to complete further brain imaging (Figures 5A-5C).

**Figure 6:**
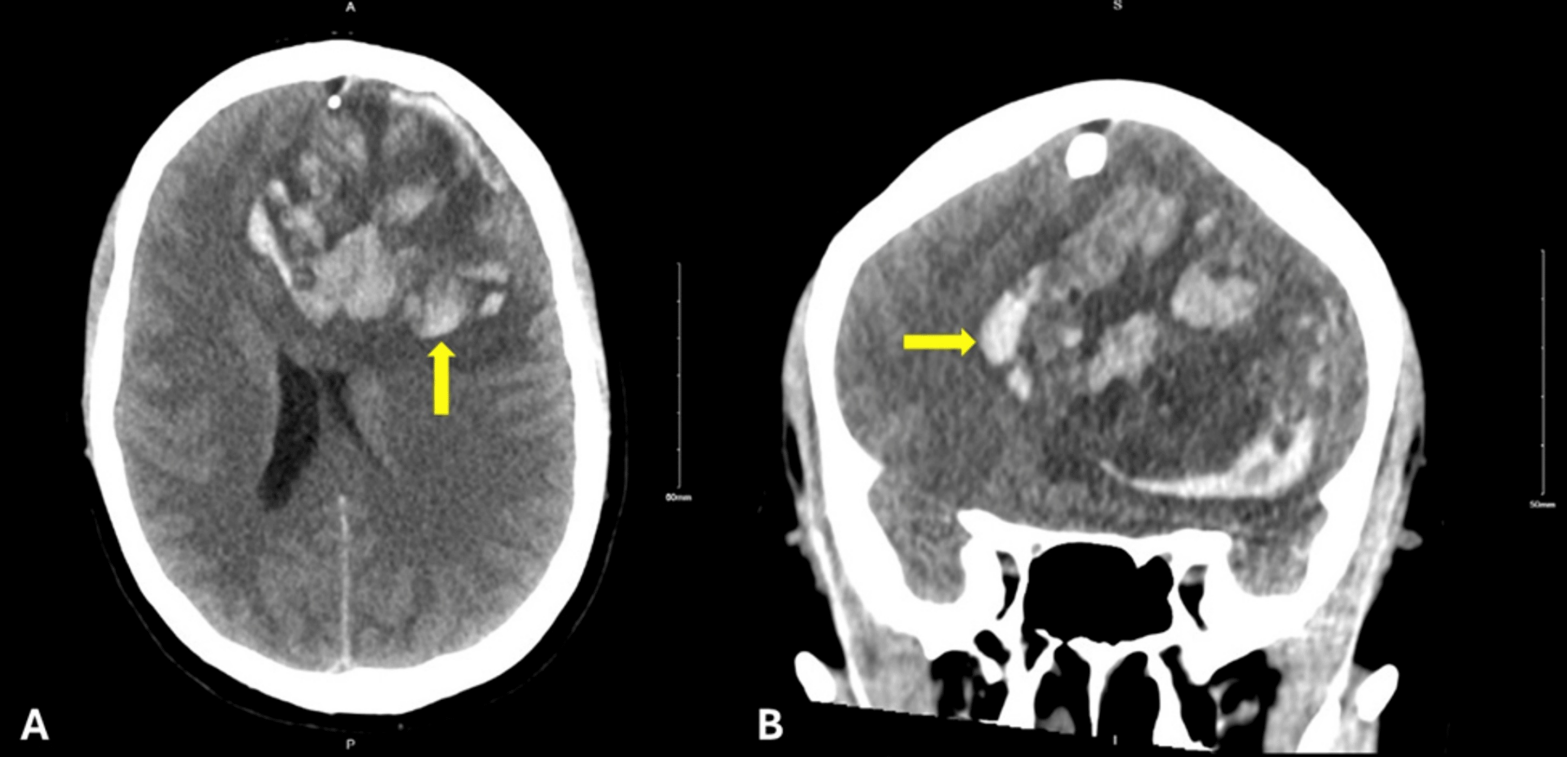
Brain metastasis with hemorrhage. Cross-sectional computed tomography imaging of the head without IV contrast. (A) Axial and (B) coronal views displaying a hemorrhagic lesion 7x7x5.5 cm (yellow arrow) in the left frontal lobe with subfalcine herniation, left uncal herniation, and midline shift.

The same evening after the PET scan was completed, the patient had multiple bouts of emesis at home, and 3 h later she was found down by family and noted to be unresponsive and posturing with agonal respirations. She was taken to an outside hospital where the examination noted a Glasgow Coma Scale (GCS) score of 3, fixed and dilated pupils, and the patient was subsequently intubated. CT scan of the head revealed a 7x7x5.5 cm hemorrhagic lesion in the left frontal lobe with subfalcine herniation, left uncal herniation, and midline shift (Figures 6A, 6B).

Neurosurgery declared that the patient’s catastrophic intracerebral hemorrhage, presumably secondary to brain metastasis, was not compatible with life. Brain death was confirmed by two-physician examination, the patient was extubated, and asystole was confirmed. The patient died at the age of 36 years and 10 months after her mastectomy.

## Discussion

We describe a patient with a five-year history of recurrent fibroadenomas in the right breast post-multiple excisions who was ultimately diagnosed with a malignant phyllodes tumor (PT), underwent right mastectomy, and eventually developed metastasis to the lung, liver, and brain nine months after mastectomy.

The histologic difference between fibroadenoma and PT can be a difficult distinction to make [1], and because both fibroadenoma and PT can carry a high degree of intralesional heterogeneity, diagnosing these on core needle biopsy alone can be misleading, as the core biopsy may not encapsulate all histologic aspects of the mass [7]. Literature shows that fibroepithelial lesions of the breast diagnosed on core needle biopsy, which also include features of stromal overgrowth or necrosis, are more commonly diagnosed as PT on final pathology after excision [8]. Additionally, recent evolutionary pathways have been identified in instances of PT with a previous history of fibroadenoma showing mutations in MED12, TERT, TP53, RARA, and PIK3CA in tumor progression [9]. The patient described in the present case was diagnosed with a malignant PT after undergoing excisional biopsies on two separate occasions for breast masses with final pathology describing fibroadenoma with necrosis. This raises the following two distinct possibilities in the present case: the patient’s previous masses were initially misdiagnosed as benign fibroadenomas, or the previously existing fibroadenomas underwent clonal evolution progressing to a higher-grade phenotype. Because both of the two previous breast masses involved pathology describing fibroadenoma with necrosis, and final pathology after the third excisional biopsy described fibroepithelial lesion with necrosis in one mass as well as phyllodes tumors in the others, it is quite possible that the subtle histologic differences between benign fibroadenoma and phyllodes tumors were overlooked on the patient’s pathology analysis of the previous two masses initially diagnosed as fibroadenoma. With the understanding that it can be difficult to distinguish the subtle histological differences between fibroadenoma and PT [1], and the presence of necrosis within a fibroepithelial lesion should raise suspicion of the presence of a PT, an area of improvement in the approach to this case would include a more extensive pathologic sampling of this patient’s previous excisions [8]. Given the known tumor heterogeneity and overall diagnostic uncertainty involved in fibroepithelial and PT masses, we recommend maintaining a heightened suspicion and utilizing additional sampling methods when evaluating fibroepithelial lesions with high-risk features, including hypercellularity, necrosis, or recurrence. In certain facilities, additional sampling methods may require the utilization of an outside pathologist with more experience in recognizing the subtle differences between fibroepithelial masses and phyllodes tumors.

There are a few case reports published describing a diagnosis of PT in patients with a previous history of fibroadenoma in the same location [10-12]. While some cases describe the emergence of PT after a fibroadenoma was diagnosed by core needle biopsy and monitored without surgery [10,11], another case describes a PT diagnosed after multiple excisional biopsies were performed for fibroadenomas [12]. Only one of these cases noted that the reported patient was alive at the time of publication [12]; however, the other two reports did not explicitly declare if the reported patients survived at the time of publication [10,11]. While there is no clear documented incidence of the diagnosis of PTs in patients with a previous history of fibroadenoma, literature shows that patients with PT who also have a history of previous fibroadenoma have better disease-free survival and overall survival compared to those with PT who do not have a prior history of fibroadenoma [13,14]. However, our patient with malignant PT and a history of previously fibroadenoma was diagnosed with metastasis to the lung, liver, and brain nine months after mastectomy, which may further support that the previous diagnoses of benign fibroadenomas in this case may have been erroneous. Literature has shown that patients with malignant PT and distant metastasis tend to have a worse overall prognosis, and similarly, our patient with metastasis died 10 months after her mastectomy [3].

Currently, surgical excision remains the cornerstone of PT treatment. While wide local excision with at least 10 mm margins is recommended for borderline and malignant PTs, more radical treatment, such as mastectomy, can be offered when adequate margins cannot be achieved [4]. At this time, the role of adjuvant radiation therapy in PT remains controversial. While the NCCN guidelines suggest that radiation may be considered in cases of malignant PT with positive margins or in recurrent disease, the supporting evidence is limited and is mostly based on retrospective series [4]. However, a recent retrospective study completed in 2021 included 108 patients with PTs and found that patients with borderline and malignant PTs who underwent mastectomy and adjuvant radiation had no significant improvement in local recurrence-free survival compared to those who did not receive adjuvant radiation. This study, however, was unable to draw any conclusions regarding distant recurrence [6]. In our presented case, the findings from Boutrus et al. were taken into consideration, and ultimately, the shared decision was made to forego adjuvant radiation [6]. The emergence of distant metastasis to the lung and presumably the brain in the present case raises the question of whether our patient might have benefited from adjuvant radiation; however, more research will need to be carried out to further evaluate distant recurrence outcomes after mastectomy in the setting of adjuvant radiation.

Malignant PTs carry a distant metastasis rate of about 20% and an extremely poor prognosis with mortality rates reported between 50% and 100%, with time of death reportedly 1.0-41.1 months after distant metastasis diagnosis [3]. The most common site of distant metastasis is the lung, with the second most common site in bone [3,15]. Literature also reports the time to develop metastasis anywhere between two months and over a decade after treatment, and of the patients who do not survive, time to death is reported to occur 1.0-41.1 months from the diagnosis of distant metastasis [3]. A retrospective study carried out in 2018 evaluated 83 patients with malignant PTs and found that large tumors (>90 mm) and tumors with malignant heterologous elements had significantly increased instances of distant metastasis [15]. Our case describes a patient who ultimately underwent mastectomy for a 95 mm malignant PT with no malignant heterologous elements identified, who subsequently developed symptoms of pulmonary metastasis nine months after mastectomy and died one month after the diagnosis of distant metastasis. Our case raises important considerations regarding risk-reduction strategies and surveillance. While PTs are not typically associated with inherited cancer syndromes, several genetic alterations have been detected in PTs, including mutations in the TERT promoter region, MED12, EGFR amplification, and TP53 alterations, suggesting a possible genetic component [16,17]. It is currently recommended that patients with findings suspicious for borderline and malignant PT undergo genetic testing if the patient is at risk for hereditary cancer syndromes [4]. Though the patient in our case never underwent hereditary genetic testing, she did elect to proceed with prophylactic contralateral mastectomy given her strong family history of cancer and recurrent breast lesions. This case also warrants discussion regarding surveillance. Given the high-risk of local recurrence, guidelines suggest that patients with borderline and malignant PTs who have undergone breast conserving surgery receive “heightened imaging” to include annual mammograms (when age appropriate), ultrasound every six months for two years, and then ultrasound annually through five years to evaluate for local recurrence; however, there are no available surveillance guidelines for patients who have undergone mastectomy for borderline and malignant PT [4]. Our case emphasizes the need for further research and definitive management guidelines in the treatment of patients with borderline and malignant PT who have undergone mastectomy, as these patients still have a high risk of distant metastasis.

## Conclusions

The distinction between a fibroepithelial tumor and phyllodes tumor of the breast is clinically significant, as phyllodes tumors and fibroadenomas differ in their biological behavior and thus their management. Malignant phyllodes of the breast is a rare and extremely aggressive malignancy with high rates of distant metastasis, which carries a poor prognosis, and these tumors pose unique diagnostic challenges due to their high degree of intralesional heterogeneity. Due to the rarity of phyllodes tumors, in particular malignant phyllodes, literature evaluating this pathology and its outcomes is sparse, and no consensus exists regarding adjuvant therapy or surveillance post-mastectomy. Our case describing rapid metastasis despite negative margins underscores the need to maintain heightened suspicion and utilize additional sampling methods when evaluating fibroepithelial lesions with high-risk features, highlights the importance of providing close follow-up for patients with recurrent fibroadenomas, and emphasizes the need for definitive surveillance and management protocols in the treatment of patients with borderline and malignant phyllodes tumors who have undergone mastectomy, as these patients still have a high risk of distant metastasis.
